# The Effect of Vitamin D_3_ on the Alignment of Mandibular Anterior Teeth: A Randomized Controlled Clinical Trial

**DOI:** 10.1155/2022/6555883

**Published:** 2022-02-14

**Authors:** Ali Al-Attar, Mushriq Abid

**Affiliations:** Department of Orthodontics, College of Dentistry, University of Baghdad, Baghdad, Iraq

## Abstract

**Objectives:**

To investigate the effect of vitamin D_3_ level on the alignment of mandibular anterior teeth in adults and explore the associated root resorption and pain perception. *Trial Design*. Multicentre, double blinded randomized clinical trial. *Subjects and Methods*. Adult patients aged 18–30 years, with moderate mandibular incisor crowding [Little's Irregularity index (LII) 3–6 mm], needing nonextraction treatment with fixed orthodontic appliance, were randomly allocated into two groups with 1 : 1 allocation ratio. In the 1^st^ group (normal vitamin D_3_ level group [ND_3_G]), vitamin D level was measured and corrected to normal before starting orthodontic treatment, while in the 2^nd^ group [control group (CG)] the vitamin D level was kept unknown until completion of the alignment phase. Outcome measures included mandibular incisor crowding using LII, orthodontically induced root resorption (OIRR), and pain perception. Independent sample *t*-test was used to compare the duration of treatment, the effectiveness of alignment, and OIRR between groups, while differences in pain perception were analysed by Mann–Whitney *U*-test (*P* < 0.05).

**Results:**

Out of 87 patients recruited from four centres, 33 patients were randomly allocated into two groups (17 patients to ND_3_G and 16 patients to CG). Time elapsed for the complete alignment of the mandibular incisor crowding was one month shorter in ND_3_G (23.532% faster), and the improvement percentage was significantly higher in all periods when compared to the CG. The amount of OIRR was not significantly different between groups; however, pain during the first three days of alignment was significantly less in ND_3_G.

**Conclusions:**

Having optimal vitamin D_3_ level reduced the alignment time and pain associated with orthodontic treatment, but it had no role in reducing OIRR. *Registration*. The trial was registered with ClinicalTrials.gov on 12^th^ April 2021 (registration number: NCT04837781).

## 1. Introduction

Orthodontic tooth movement (OTM) occurs as a consequence of applying force due to the periodontal ligament and alveolar bone remodelling, as a result of local alterations in blood flow which stimulate the release of various inflammatory mediators [1]. Long treatment times have remained one of the most significant problems in orthodontic therapy. Comprehensive orthodontic treatment with fixed appliances may take an average of two years [2], which may be extended depending on the severity of the malocclusion, the difficulty of the procedure, or clinician and patient considerations [3]. Longer treatment times may be detrimental to both orthodontists and patients, as it might expose the patient to the possibility of decay, OIRR, and periodontal problems [4–6]. As a result, minimizing treatment time has been a top priority for both orthodontists and patients [7]. In an attempt to reduce treatment duration, several surgical and nonsurgical methods were introduced which aim to increase bone remodelling and thus accelerate tooth movement [8]. Among the nonsurgical approaches, vitamin D_3_ caused an increase in osteoclast activity resulting in accelerated OTM [9].

Vitamin D_3_ is a fat-soluble vitamin normally found only in a few foods, added to some, and provided as dietary supplements. It is also endogenously produced when ultraviolet rays from sunlight reach the skin and cause vitamin D_3_ synthesis. Vitamin D_3_ is biologically inactive and requires two hydroxylation reactions to become active in the body. The first takes place in the liver and transforms vitamin D to 25-hydroxyvitamin D [25(OH) D], also referred to as calcidiol. The second reaction happens predominantly in the kidney and forms 1,25-dihydroxyvitamin D [1, 25(OH)_2_ D], also described as calcitriol, which is the physiologically active form of vitamin D_3_ [10].

The best measure of vitamin D_3_ status is serum concentration of 25(OH) D. It represents vitamin D_3_ generated cutaneously, as well as being obtained from diet and supplements, and has a comparatively long half-life of 15 days. Unlike 25(OH) D, circulating 1,25(OH) 2D is usually not a good predictor of vitamin D_3_ status, since it has a brief 15-hour half-life and serum concentrations are strongly controlled by parathyroid hormone (PTH), calcium, and phosphate. 1,25(OH)_2_ D levels are not usually reduced until vitamin D_3_ deficiency is severe [11].

An association has been discovered between vitamin D_3_ receptor polymorphisms and periodontitis and bone metabolism [12]. Researchers found that vitamin D_3_, parathyroid hormone, and calcitonin all regulate calcium and phosphorus levels [13]. Vitamin D_3_ promoted bone resorption by causing osteoclast differentiation from their progenitors and by enhancing existing osteoclast activity [14, 15].

Animal studies reported an increased rate of OTM following a local injection of vitamin D_3_ when compared to the contralateral side [16, 17]. Effect of vitamin D_3_ on OTM in humans was rarely investigated, the earlier attempt in this field was done by Al-Hasani et al., in 2011, which investigated the effect of local application of calcitriol (active form of vitamin D_3_) on OTM; their results revealed nonsignificant variations between the control and experimental side when different doses were used [18]. A recent split-mouth RCT, which examined the clinical and radiographic effect of locally administered calcitriol on canine distalization, revealed a significant increase in the rate of canine distalization and a decline in the bone density on the experimental side relative to the control side [19]. The relationship between vitamin D_3_ deficiency and OIRR is contradicting; while some authors found a positive relation [20], others reported no relation [21].

A recently published cumulative systematic review and meta-analysis suggested that calcitriol could accelerate OTM in both animals and humans. However, the studies were carried out using inadequate sampling and supported by a low level of evidence [22]. No previous study investigated the effect of optimizing vitamin D_3_ level on the rate of mandibular incisors crowding, OIRR, and pain perception. Therefore, this study was planned to investigate the effect of optimizing the vitamin D_3_ level on the duration of mandibular incisor alignment treatment. The primary objective was to compare mandibular incisor crowding during alignment, at 4-week interval from the start of treatment. The secondary objectives were to compare the amount of OIRR in the mandibular incisor apical region and to compare pain perception during the first week of orthodontic treatment.

The null hypothesis is that “there is no effect of optimizing vitamin D_3_ level on the duration of treatment during the initial phase of orthodontic treatment.”

## 2. Subjects and Methods

### 2.1. Trial Design

The study was a multicentre randomized controlled clinical trial, double blinded designed and parallel groups with equal allocation ratio (1 : 1 ratio). There were no changes to the method after trial commencement. The trial was registered with ClinicalTrials.gov on 12^th^ April 2021 (registration number: NCT04837781).

### 2.2. Participants

Participants involved patients that needed fixed appliance orthodontic treatment according to the following criteria.

#### 2.2.1. Inclusion Criteria

1. Adult patients aged 18–30 years
2. Moderate lower incisor crowding (Little's irregularity index (LII): 3–6 mm)
3. Requirement of nonextraction treatment in the mandibular arch
4. Full set of permanent dentitions excluding the third molar
5. Good oral hygiene and no periodontal disease
6. No history of root resorption or trauma to the mandibular anterior teeth

#### 2.2.2. Exclusion Criteria

1. Medically compromised patients
2. Patients with thyroid, parathyroid, or renal and liver disease
3. Patients on medication, especially corticosteroid and anticonvulsant drugs
4. Poor oral hygiene

The trial was performed in the Orthodontic Clinic at the College of Dentistry, University of Baghdad, in addition to two specialized governmental dental centres and one private clinic in Baghdad city, Iraq. Ethical approval was granted from the Ethics Committee of the College of Dentistry, University of Baghdad, on 16^th^ January 2020 with ID number: 178420.

The primary investigator (A. A.) firstly assessed the participants for eligibility in the study; those who met the inclusion criteria were notified to get primary acceptance for contribution. Then, the patient information sheets were provided which explained the nature of the study in the form of a series of questions and answers. The consent form was signed by the participants.

Participants were divided blindly into two groups. In the first group (normal vitamin D_3_ level group (ND_3_G)), vitamin D_3_ level was measured before orthodontic intervention; if the level was below the normal value (30 ng/mL), then the participants were referred to an endocrine specialist to optimize the level of vitamin D_3_ to normal before bonding of the appliance. While in the control group (CG), orthodontic treatment was performed without measuring the level of vitamin D_3_ until completion of the alignment phase (Supplemental Table 1). At the time of measuring vitamin D_3_ level, parathyroid hormone (PTH), serum calcium (Ca), serum phosphate (P), and serum albumin were also measured.

### 2.3. Interventions

All the participants received the same standardized treatment protocol. After profound teeth polishing with nonfluoridated pumice, and etching with 37% phosphoric acid, brackets (Pinnacle®, MBT prescription with 0.022-inch slot, Ortho Technology, Champaign, Illinois, USA) were bonded with light-cured adhesive paste (3M™ Transbond™ XT, USA), using a bracket height gauge for standardization. The arch wire sequence of 0.016-inch, 0.016 × 0.022-inch, and 0.019 × 0.025-inch heat activated nickel titanium (HANT) (TruFlex^TM^ Thermal Ortho Technology, Champaign, Illinois, USA) was used for alignment [23]. An initial 0.016-inch HANT wire was placed on the bonding day and fully tied to the bracket by elastomeric modules. When the expected improvement was achieved and the next wire could be inserted with minimal deflection during full ligation, arch wire was replaced. The alignment was considered to be finished when a working arch wire of 0.019 × 0.025-inch stainless steel (TruForce^TM^ Ortho Technology, Champaign, Illinois, USA) could be placed passively. In case of bracket debonding during treatment, the participants were informed to call and arrange an emergency appointment within 24 hours for rebonding; otherwise, the case would be considered as a dropout.

An alginate impression (Lascod, Millenium®, Italy) for the mandibular arch was taken at pretreatment (T0) and then every four weeks until the alignment was finished. After pouring the impressions with Type 4 extra-hard Dental Die Stone (Lascod, Singletypo4®, Italy), a study model was obtained. The study model was scanned with a 3D scanner (Smart Optic Vinyl, Bochum, Germany) to produce a 3D digital model on which all measurements were taken using the AutoCAD® 2020 (https://www.autodesk.co.uk) software program. Digital Periapical radiographs were taken for the mandibular incisors at pretreatment (T0) and after 12 weeks (T3) for both groups, using a long cone paralleling technique. Furthermore, a Visual Analogue Scale (VAS) was provided to the participants to record their pain perception after wire placement during the first week.

### 2.4. Primary Outcome (Alignment Efficiency)

Little's irregularity index (LII) [24] was measured on a digital model to calculate the amount of contact displacement mesially and distally from the mesial contact point of the lower left to the right canines. When a 0-1 mm irregularity index was accomplished, and the alignment improvement did not exceed 0.5 mm between two successive visits, then the alignment phase was considered to be completed.

### 2.5. Secondary Outcomes

#### 2.5.1. Root Resorption

Root resorption was evaluated before treatment (T0) and after 12 weeks (T3) for both groups by periapical radiograph using digital sensor (Nanopix 2, Eighteeth, Jiangsu, China), which was positioned by sensor holder for mandibular incisors, using a long cone paralleling technique with a 7 cm film-cone distance. The radiographic machine (Runyes®, Model: Ray68(M), Ningbo, China) was set to 70 kV voltage and 8 mA amperage with an exposure time was of 0.25 seconds.

**Figure 1:**
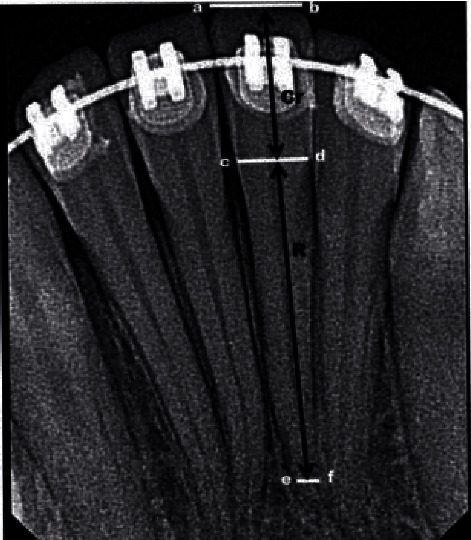
Root length measurement (a) mesial incisal edge point; (b) distal incisal edge point; (c) mesial cementoenamel junction point; (d) distal cementoenamel junction point; (e) mesial edge point of the apical foramen; (f) distal edge point of the apical foramen; (Cr) crown length; (R) root length.

OIRR of lower incisors was assessed according to the following method. First, several mesial and distal points (a, b, c, d, e, and f) at incisal edge, cementoenamel junction, and apical foramen, respectively, on pre- and posttreatment radiographs were appointed. Then, horizontal lines (a-b, c-d, and e-f) were drawn to connect points. The vertical distance between the centre of the lines a-b and c-d represented crown length “Cr,” while the vertical distance between the centres of the lines c-d and e-f represented root length “R.” Crown length was used as reference to overcome possible dimensional error between pre- and posttreatment radiographs (Figure 1).

The means of the pre- and posttreatment crown lengths were measured according to the following equation: Crx = (Cr1 + Cr2)/2, where Crx = average crown length, Cr1 = pretreatment crown length, and Cr2 = posttreatment crown length.

Then, the corrected root length in pre- and posttreatment was measured using the following formulae:

 
*R*_*c*1_(corrected) = *R*1 × (Crx/Cr1), where *R*1 = pretreatment root lengths
 
*R*_*c*2_(corrected) = *R*2 × (Crx/Cr2), where *R*2 = posttreatment root lengths

The difference between Rc1 and Rc2 represented the amount of root resorption [25].

#### 2.5.2. Pain Perception

Over the first 7 days after bonding, pain was assessed every day in the evening using a 10-point Visual Analogue Scale [VAS] (Supplementary Figure 1). Each patient described the most severe pain he/she had ever felt. On the day of bonding, all patients received the recording sheet, which included seven VASs (one for each day), and we instructed patients on how to fill the VAS by encircling the point on the line that was felt to characterize the maximum pain that they sensed per day, with 0 referring to “no pain” and 10 referring to “unbearable pain.” Patients were prompted to label the recording sheet and return it in their next appointment via phone call on a daily basis.

There were no outcome changes after the trial had begun. A summary of data collection is demonstrated in Supplementary Table 2.

### 2.6. Sample Size Calculation

The sample size was calculated using power analysis (*G*^*∗*^ Power 3.1, Aichach, Germany) [26]. Full alignment of the lower anterior teeth in patients with moderate crowding treated without extractions takes about 117 days [27]. The sample size was calculated to identify a significant mean difference of 28 days in overall alignment and leveling time with a standard deviation of 15.29 [28]. A minimum of 11 participants were required in each group, using 95% statistical power and 0.01 alpha-level. The sample was increased to 15 per group to overcome dropouts.

### 2.7. Interim Analyses and Stopping Guidelines

If there was severe pain or root resorption in the mandibular incisors for any participant in each group, then the trial should be terminated.

### 2.8. Randomization

To build basic randomization and ensure an equal 1 : 1 allocation ratio, computer software program (https://www.graphpad.com/quickcalcs/randomn2.cfm) was used to generate random numbers. To ensure allocation concealment, identical opaque sealed envelopes were employed. An independent person assigned a study number to each number in the resulting random table in order to create the allocation table. Because it is the sole document that may unmask the groups, the allocation table was kept hidden from the clinical team until the data measurement and analysis were completed. At the time of bonding up the fixed appliance, an independent dental staff shuffled the concealed envelopes, and the participant was asked to pick one envelope; thus the investigator (assessor) was not involved in the randomization process.

### 2.9. Blinding

Clinicians and investigator (data analyser) were blind to treatment allocation but, due to the nature of the study, the participants in the first group knew their level of vitamin D_3_ from the beginning, so it was impossible to blind the participants. The research ID number was labelled on all trial documents and was used to identify participants and gather data without revealing the allocation group. This allowed the investigator to gather data and make measurements blindly.

### 2.10. Statistical Analysis

Statistical Package for Social Sciences, version 25.0 (SPSS Inc., Chicago, Illinois, USA) was used for data analysis. The following statistical assessments were used: Descriptive statistics included number, percentages, mean, and standard deviation. Meanwhile, reliability statistics included intraclass correlation coefficient (ICC) was used to test inter- and intraexaminer reliability for Little's irregularity index of ten study models and root length of lower incisors for ten periapical radiograph measured twice with a 4-week interval.

#### 2.10.1. Inferential Statistics

The data was found to be normally distributed when examined by Shapiro–Wilk test; therefore, parametric tests were used, which included the following:

- Independent sample *t*-test: to compare treatment duration time of lower anterior teeth alignment completion between groups, in addition to alignment efficiency at various time intervals, alignment improvement percentage, and amount of root resorption between groups
- Paired *t*-test: to compare root length between pre- and posttreatment radiographs within the same group
- Mann–Whitney *U*-test: adopted to test pain perception score between groups

The significance level was set as *P* < 0.05.

## 3. Results

Out of 87 patients who were assessed for eligibility, only 42 patients fit the inclusion criteria and nine of them declined to participate in the study. Therefore, 33 patients were finally enrolled in the trial, and they were randomly divided into two groups (ND_3_G and CG). Patients were recruited from the Orthodontic Department, College of Dentistry, University of Baghdad, in addition to two specialized governmental dental centres and one private clinic in Baghdad city.

**Figure 2:**
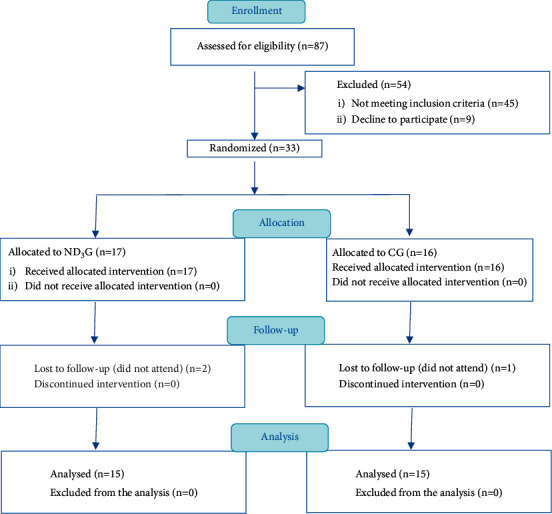
CONSORT diagram showing the flow of subjects through the trial.

The mean age of participants was 20.13 ± 1.81 years. The CONSORT flowchart of the participants for this trial is illustrated in Figure 2. Participant recruitment occurred between January 2020 and November 2020 and the trial was completed as scheduled.

Even with the adequate sample size in the current trial (within the central limit theorem, ≥30) [29, 30], Shapiro–Wilk test was used to assess the normal distribution of the data and it was found to be normally distributed (*P* > 0.05). Baseline characteristics of participants are shown in Table 1.

An ICC shows high reliability when measuring LII [intraexaminer: 0.98 (95% CI: 0.94–0.99), interexaminer: 0.97 (95% CI: 0.90–0.99)] and lower incisor root length (intraexaminer: 0.99 (95% CI: 0.93–0.99), interexaminer: 0.98 (95% CI: 0.93–0.99)).

Thirty-three was the total number of participants in this trial (17 patients in ND_3_G and 16 patients in CG), two patients in ND_3_G and one patient in CG were lost to follow-up, so final analysed patients were 30 (15 in each group). There were no qualitative variations in each centre's patient pool because they were all selected based on predefined criteria. Additionally, no centre-effects were found in the trial since all specialists strictly followed the research protocol.

### 3.1. Alignment Efficacy

**Figure 3:**
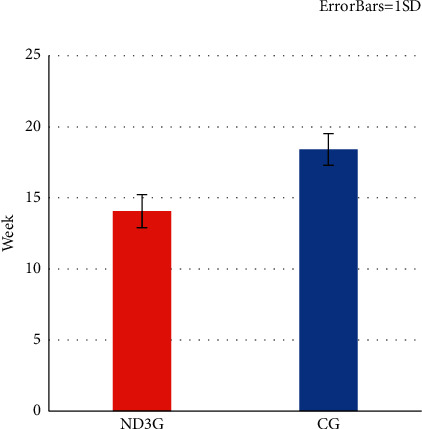
Duration of treatment.

**Figure 4:**
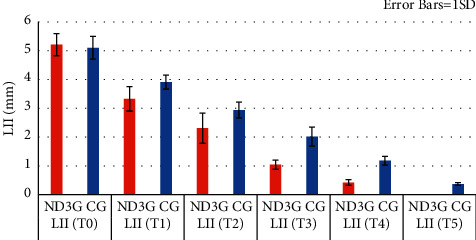
Amount of little irregularity index at various time interval.

The total time needed to resolve lower anterior teeth crowding was one month shorter in ND_3_G (Table 2) and (Figure 3), so treatment time was 23.5% faster in ND_3_G when compared to CG. Despite the nonsignificant differences in LII at the start of treatment between groups, there was a significantly higher improvement in reduction of crowding in ND_3_G at all treatment intervals stage (Table 3 and Figure 4). Thus, alignment improvement percentage (calculated by dividing the LII value amount at a specific time point (calculated by subtracting the LII value at T1, T2, T3, and T4 from the LII value at T0) by LII value at T0) was higher in ND_3_G during various time intervals (Table 4), resulting in faster treatment time by 1.59 at T1, 1.34 at T2,1.33 at T3, and 1.2 at T4 in ND_3_G.

### 3.2. Root Resoprtion

No significant difference was found between the groups, although there was a significant reduction in root length after 12 weeks of alignment compared to baseline pretreatment root length for both groups (Table 5).

### 3.3. Pain

Pain score was higher in CG with a significant difference (*p* < 0.05) for the first three days and then gradually subsided with no significant difference between groups (Table 6).

### 3.4. Harms

Except for mild discomfort and root resorption, which did not exceed what normally happens during orthodontic treatment, there were no serious adverse events recorded during the research.

## 4. Discussion

The goal of this study was to investigate the effect of optimizing the level of vitamin D_3_ on alignment efficiency, root resorption, and pain perception during the early phase of orthodontic therapy. The study was conducted as a multicentre randomized clinical trial. There were statistically significant differences between ND_3_G and CG, according to the findings. As a result, the null hypothesis was rejected.

Based on the patient allocation among centres, providing that there were no qualitative variations in each centre's patient pool and that a stringent research procedure was followed, this might reduce the degree of unequal distribution between centres. The sample size (30 individuals) yielded a power of 80%. This overall sample size was comparable to other clinical trials with similar objectives, which had sample sizes ranging from 25 to 35 [31–33].

The baseline characteristics showed no significant difference in the age of the participants, which were selected in a range of 18–30 years to overcome the effect of growth on OTM. LII measurement was between 3 and 6 mm, as such crowding could be solved without extraction to minimize the effect of confounding factors.

The dependent variable (vitamin D_3_ level) being higher in ND_3_G was intentionally corrected to reach normal level before starting orthodontic treatment, while in the CG vitamin D_3_ level was unknown until completion of the alignment phase. However, when measured, it was found to be low in the majority of participants (12.28 ng/mL ± 5.2). Keeping in mind that vitamin D_3_ deficiency is a worldwide problem [34–38], this group reflected the actual vitamin D_3_ level in the general population. Although serum calcium and phosphate level were low in the CG, they were still within the normal range. Several investigators found a balanced level of Ca and P in spite of low levels of vitamin D_3,_ due to the higher level of parathyroid hormone which stimulates calcium and phosphate reabsorption from kidneys [39–42].

In the present study, PTH, Ca, P, and serum albumin were measured in addition to vitamin D_3_ levels, since they provide an indication regarding activity of vitamin D_3_; PTH upregulates the activity of 1-*α*-hydroxylase enzyme, which converts 25-hydroxycholecalciferol, the major circulating form of inactive vitamin D_3_, into 1,25-dihydroxycholecalciferol, the active form of vitamin D_3_, in the kidney. If blood calcium and/or phosphate levels were low and 25-hydroxycholecalciferol was normal, this might indicate a problem in the activation of vitamin D in the kidney. Finally, most laboratories measure total calcium in serum, which needs to be corrected if serum albumin is below 4.0 g/dL as follows: Ca (mg/dL) = measured Ca (mg/dL) + 4.0 –Alb (g/dL) [43].

Our trial reported a shorter time needed to align the mandibular incisors in ND_3_G and higher improvement percentage of the alignment at various time intervals. The study supported the effect of vitamin D_3_ in increasing bone remodelling, thus accelerating OTM. This is in accordance with previous studies that reported an increase in the rate of OTM and decrease in bone density following local injections of the active form of vitamin D_3_ (calcitriol) [19, 44]. Conversely, Al-Hasani et al. reported no significant difference between the control and experimental side after local calcitriol injection distal to the upper canine, which could be due to the very short time of trial (3 weeks) and small sample size (5 per group) [18].

The effect of vitamin D_3_ on bone remodelling is emphasized by the effect of the active form (calcitriol) on bone metabolism, the presence of its receptor on osteoblasts, osteoclast precursors, and osteoclasts, and the fact that it enhances bone turnover by stimulating prostaglandin production in osteoblasts [45]. Furthermore, vitamin D_3_ regulates the production of collagen type I alkaline phosphatase, osteocalcin, and osteoblastic proliferation [46]. The active form of vitamin D_3_ (calcitriol) stimulates bone marrow to form osteoclasts, so it acts as an inducer of osteoclastic bone resorption [47].

Some unexplored variables have a significant influence on oral environment such as use of probiotics [48] and natural compounds [49]; therefore, they could have an effect also in combination with vitamin D_3_ on tooth movement.

The crown and root lengths of the four lower incisors were measured, and a correction factor was utilized. This method is more reliable for assessing root resorption following orthodontic treatment when compared to the scoring method, which is subjective. Digital periapical X-ray imaging was used, since it was less invasive to patients than 3D radiographs.

There were significant differences between pre- and posttreatment root length in both groups, but within normal limits of root resorption following OTM, as reported by other studies [31, 50, 51]. There was no significant difference between the groups regarding root resorption, which agrees with Tehranchi et al., who suggested that vitamin D level was not among the clinical variables that are potential contributors for OIRR [21].

VAS was used to assess pain perception during the first week after insertion of the first aligning wire. The average score of pain was less in the three first days in the ND_3_G when compared to the CG during the same period. This result may open the way to a recent method of treatment for pain by vitamin D_3_ supplement [52–55], since studies found inverse relation between vitamin D_3_ level and pain level [56, 57].

### 4.1. Limitations

This study was conducted on adult patients (18–30 years) to overcome the effect of growth. However, more studies are needed to investigate the effect of vitamin D_3_ on OTM in adolescents, with a larger sample size, and evaluate inflammatory markers during treatment via gingival crevicular fluid. The present study used digital periapical radiographs to ensure a safer radiation dose, but cone beam computerized tomography provides a better evaluation of root resorption. It was impossible to blind participants to the allocation group due to the nature of the study, but they were strictly blinded to the allocation group table.

Notably, the aims of the current study were met with little influence from the aforementioned limitations, because they were minor and had no effect on the primary or secondary outcomes.

## 5. Conclusions

The duration of lower incisor alignment therapy was shorter in the group with normalized vitamin D_3_ level, and the percentage of alignment improvement was significantly higher in this group during the various stage of treatment. Vitamin D_3_ plays a role in reducing pain associated with OTM, but had no effect in reducing orthodontically induced root resorption.

## Figures and Tables

**Table 1:** Demographic characteristic of patients.

Variable	Total	ND_3_G	CG	*P*-value	MD [95% CI]
	Mean (SD)	Mean (SD)	Mean (SD)		
Age (years)	20.13 (1.81)	20.5 (1.61)	19.70 (1.95)	0.197	0.866 [−0.47–2.208]
Starting little irregularity index (mm)	5.15 (0.38)	5.21 (0.38)	5.10 (0.39)	0.432	0.11 [−0.17–0.40]
Vit.D (ng/ml)	23.17 (11.81)	34.06 (2.78)	12.28 (5.21)	*P* ≤ 0.001	21.78 [18.65–24.91]
PTH (pg/ml)	46.66 (13.50)	37.73 (7.29)	55.60 (12.39)	*P* ≤ 0.001	−17.86 [−25.47–−10.26]
Calcium (mg/dl)	8.38 (0.77)	9.11 (0.37)	8.06 (0.70)	*P* ≤ 0.001	1.053 [0.63–1.47]
Phosphate (mg/dl)	3.57 (0.60)	3.90 (0.55)	3.24 (0.44)	0.001^*∗*^	0.666 [0.28–1.04]
Albumin (g/dl)	4.18 (0.27)	4.26 (0.21)	4.09 (0.31)	0.08	0.17 [−0.02–0.37]

CI = confidence interval; MD = mean difference; ^*∗*^= significance at *P* < 0.05; SD = standard deviation.

**Table 2:** Mean treatment time for mandibular anterior teeth alignment.

Group	Mean (week)	SD	*P*-value	MD [95% CI]
ND_3_G	14.07	1.16	*P* ≤ 0.001	−4.33 [−5.18–−3.34]
CG	18.40	1.12		

CI = confidence interval; MD = mean difference; ^*∗*^= significance at *P* < 0.05; SD = standard deviation.

**Table 3:** Amount of crowding at various time intervals between groups.

Time interval	ND_3_G	CG	*P*-value	MD [95% CI]
	Mean (SD)	Mean (SD)		
T0	5.21 (0.38)	5.1 (0.394)	0.432	0.113 [−0.177-0.404]
T1	3.32 (0.42)	3.906 (0.240)	*P* ≤ 0.001	−0.580 [−0.839–−0.320]
T2	2.30 (0.52)	2.940 (0.272)	*P* ≤ 0.001	−0.633 [−0.947–−0.318]
T3	1.04 (0.15)	2.013 (o.331)	*P* ≤ 0.001	−0.949 [−1.167–−0.778]
T4	0.42 (0.10)	1.180 (0.152)	*P* ≤ 0.001	−0.760 [−0.856–−0.663]
T5		0.37 (0.05)		

T0 = baseline; T1 = after 4 weeks; T2 = after 8 weeks; T3 = after 12 weeks; T4 = after 16 weeks; T5 = after 20 weeks; CI = confidence interval; MD = mean difference; ^*∗*^= significance at *P* < 0.05; SD = standard deviation.

**Table 4:** Improvement percentages between groups.

Assessment point	ND_3_G	CG	*P*-value	MD [95% CI]	% Change	Time faster
(T0 − T1)/T0	35.9 (8.68)	23.251 (3.333)	*P* ≤ 0.001	12.743 [7.824–17.662]	59.38 (49.77)	1.59 (0.50)
(T0 − T2)/T0	55.60 (10.42)	42.246 (4.509)	*P* ≤ 0.001	13.357 [7.353–19.360]	34.02 (32.20)	1.34 (0.32)
(T0 − T3)/T0	80.08 (2.41)	60.546 (5.388)	*P* ≤ 0.001	19.538 [16.416–22.660]	33.08 (10.54)	1.33 (0.11)
(T0 − T4)/T0	91.94 (1.87)	76.864 (2.300)	*P* ≤ 0.001	15.074 [13.505–16.643]	19.70 (3.85)	1.20 (0.04)

T0 = baseline; T1 = after 4 weeks; T2 = after 8 weeks; T3 = after 12 weeks; T4 = after 16 weeks; CI = confidence interval; MD = mean difference; ^*∗*^= significance at *P* < 0.05; SD = standard deviation; % = percentage.

**Table 5:** *t*-test for pre- and posttreatment root length within and between groups.

ND_3_C		*P*-value	MD [95% CI]	CG		*P*-value	MD [95% CI]	Between groups			
Pre. RL	Post. RL	*P* ≤ 0.001	0.773 [0.58–0.96]	Pre.RL	Post.RL	*P* ≤ 0.001	0.893 [0.76–1.02]	Pre		Post	
Mean (SD)	Mean (SD)			Mean (SD)	Mean (SD)			*P*-value	MD [95% CI]	*P*-value	MD [95% CI]
14.50 (0.87)	13.73 (0.93)			14.30 (0.61)	13.41 (0.72)			0.477	0.20 [−0.36–0.76]	0.303	0.320 [−0.30–0.72]

CI = confidence interval; MD = mean difference; ^*∗*^= significance at *P* < 0.05; RL = root length; SD = standard deviation.

**Table 6:** Mann–Whiteny *U* test comparing pain perception for the first seven days.

Day	Group	N	Mean rank	Sum of ranks	*P-*value
1^st^	ND_3_G	15	11.43	171.50	0.010^*∗*^
	CG	15	19.57	293.50	

2^nd^	ND_3_G	15	11.73	176.00	0.019^*∗*^
	CG	15	19.27	289.00	

3^rd^	ND_3_G	15	10.93	164.00	0.004^*∗*^
	CG	15	20.07	301.00	

4^th^	ND_3_G	15	13.50	202.50	0.217
	CG	15	17.50	262.50	

5^th^	ND_3_G	15	15.00	225.00	0.775
	CG	15	16.00	240.00	

6^th^	ND_3_G	15	14.50	217.50	0.539
	CG	15	16.50	247.50	

7^th^	ND_3_G	15	15.50	232.50	1.000
	CG	15	15.50	232.50	

^*∗*^= significance at *P* < 0.05.

## Data Availability

The data used to support the findings of this study are made available from the corresponding author upon request.

## Supplementary Materials

Supplementary Materials Supplemental Figure 1: Visual Analogue Scale (VAS). Supplemental Table 1: vitamin D_3_ level in both groups. Supplemental Table 2: the data that was collected and measured during the course of this trial.
