# Supplementary material for: The Effect of Vitamin D3 on the Alignment of Mandibular Anterior Teeth: A Randomized Controlled Clinical Trial
Source: Int J Dent. 2022 Feb 14;2022:6555883. doi: 10.1155/2022/6555883 (PMC8860536; doi:10.1155/2022/6555883)
Supplement: Supplementary Materials — Supplemental Figure 1: Visual Analogue Scale (VAS). Supplemental Table 1: vitamin D3 level in both groups. Supplemental Table 2: the data that was collected and measured during the course of this trial. [file 6555883.f1.docx]

**Supplementary file**

**Supplemental figure 1: Visual Analog Scale (VAS).**

**
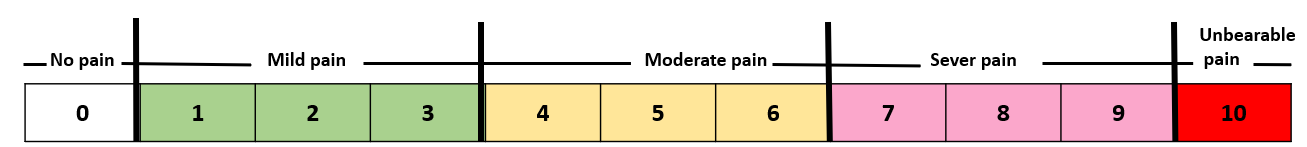
**

**Supplemental Table 1: Vitamin D_3_ level in both groups**

| **No.** | **Normal vitamin D_3_ group (ND_3_G)** | | **Control group** |
| --- | --- | --- | --- |
|  | **Initially before starting the treatment** | **After optimizing Vit.D_3_ level** |  |
| **1** | 12 | 32 | 11 |
| **2** | 18 | 34 | 9.5 |
| **3** | 12 | 31 | 7.8 |
| **4** | 19 | 33 | 13.4 |
| **5** | 9 | 32 | 23 |
| **6** | 18 | 38 | 12.2 |
| **7** | 12 | 32 | 8.4 |
| **8** | 18 | 37 | 7.3 |
| **9** | 13 | 32 | 24.6 |
| **10** | 24 | 40 | 8.2 |
| **11** | 8 | 33 | 15.6 |
| **12** | 15 | 32 | 10.4 |
| **13** | 14 | 34 | 9.6 |
| **14** | 16 | 38 | 13 |
| **15** | 12 | 33 | 10.2 |
| **Mean** | 14.66 | 34.06 | 12.28 |
| **SD** | 4.22 | 2.78 | 5.21 |

**Supplemental table 2: The data that was collected and measured during the course of this trial**

| Type of measurement | | Pre-treatment **T0** | After 4 weeks **T1** | After 8 weeks **T2** | After 12weeks **T3** | After 16 weeks **T4** | After 20 weeks **T5** |
| --- | --- | --- | --- | --- | --- | --- | --- |
| **Little’s irregularity index** | | √ | √ | √ | √ | √ | √ |
| **Root length** | | √ |  |  | √ |  |  |
| **Pain perception** | 1^st^  day | 2^nd^  day | 3^rd^  day | 4^th^  day | 5^th^  day | 6^th^  day | 7^th^  day |
